# Laparoscopic sacrocolpopexy mesh excision step-by-step

**DOI:** 10.1007/s00192-023-05494-5

**Published:** 2023-03-10

**Authors:** Stefan Mohr, Sara Imboden, Michael D. Mueller, Annette Kuhn

**Affiliations:** Department of Obstetrics and Gynecology, Inselspital, Bern University Women’s Hospital, University of Bern, Friedbühlstrasse 19, 3010 Bern, Switzerland

**Keywords:** Mesh complication, Minimally invasive mesh excision, Pelvic organ prolapse, POP, Repeat surgery

## Abstract

**Introduction and hypothesis:**

The objective was to demonstrate the surgical procedure of laparoscopic mesh removal after sacrocolpopexy to aid clinicians facing mesh complications.

**Methods:**

Video footage shows the laparoscopic management of mesh failure and mesh erosion after sacrocolpopexy with narrated video sequences of two patients.

**Results:**

Laparoscopic sacrocolpopexy represents the gold standard in advanced prolapse repair. Mesh complications occur infrequently but infections, failure of prolapse repair and mesh erosions necessitate mesh removal and repeat sacrocolpopexy if applicable.

The video deals with two women referred to our tertiary referral urogynecology unit in the University Women’s Hospital of Bern, Switzerland, after laparoscopic sacrocolpopexies that were carried out in remote hospitals. Both patients were asymptomatic more than 1 year after surgery.

**Conclusions:**

Complete mesh removal after sacrocolpopexy and repeat prolapse surgery can be challenging but is feasible and is aimed at improving patients’ complaints and symptoms.

**Supplementary Information:**

The online version of this article (10.1007/s00192-023-05494-5) contains supplementary material. This video is also available to watch on http://link.springer.com/. Please search for this article by the article title or DOI number, and on the article page click on ‘Supplementary Material’

## Introduction

Sacrocolpopexy is the preferred route for treating apical prolapse [1] since patient satisfaction and re-operation rate are favorable [2]. However, mesh complications are raising concerns as this foreign material can erode, cause pain, or become infected, and mesh complication rates are reported to occur in 0–5% [2].

Mesh exposure (69.2%) and pain (57.7%) are the most frequent indications for mesh removal [3]. After mesh excision, prolapse recurs in 46% beyond the hymen, meaning a 15-fold risk of prolapse recurrence compared with patients after sacrocolpopexy without mesh removal [3]. In patients with failed sacrocolpopexy, it is advised to identify points of mesh detachment, anatomical landmarks, removal of the prior vaginal portion of the mesh, and attachment of a new surgical mesh to either the sacrum or the sacral portion of the mesh [4].

The aim of this step-by-step video is to provide examples of mesh removal in patients with large mesh erosion and failed sacrocolpopexy, respectively, to aid clinicians facing mesh complications.

## Materials and methods

Demonstration of the laparoscopic management of mesh failure and mesh erosion after sacrocolpopexy with narrated video footage.

Patient #1 was 52 years old and had undergone a laparoscopic sacrocolpopexy in 2017. Her gynecologist found a mesh erosion of almost the entire posterior vaginal wall and a recurrent prolapse. The patient complained of intermittent vaginal bleeding and the sensation of a vaginal bulge. She had had six spontaneous deliveries and several previous abdominal operations (laparoscopic gastric bypass operation in 2013, laparoscopic inguinal hernia repair in 2015 and revision in 2016, laparotomy for internal hernia in 2015, laparotomy for perforated appendicitis in 2017, and umbilical hernia repair in 2018). Three years after the sacrocolpopexy we removed the whole mesh from the posterior vaginal wall. No mesh was found to be attached to the vaginal apex. We sutured the posterior vaginal wall. We did a repeat sacrocolpopexy 4 months after mesh removal as the patient complained of progressive prolapse symptoms (feeling of a foreign body in the vagina) with a POP-Q 0/0/−4, 3/3/8, 0/1/−4. The new mesh was placed in the same way in which we routinely apply mesh during primary laparoscopic surgery down to the bladder neck and deep into the pouch of Douglas, but without fixation to the pelvic floor muscles.

Patient #2 was 51 years of age. Her history revealed that she had undergone a vaginal hysterectomy with anterior and posterior colporrhaphy and sacrospinous ligament fixation in 2012 and a laparoscopic sacrocolpopexy in 2019. She was referred because of immediate recurrent prolapse (cystocele POP-Q grade 2, apical prolapse POP-Q 1) after this operation. Cystoscopically and during vaginal examination no mesh erosion was found. Ten months after the initial sacrocolpopexy we excised the dislocated mesh from the promontory and the right lateral pelvic wall. A repeat sacrocolpopexy was carried out during this same operation because the patient experienced a strong foreign body and tearing sensation in the vagina at a POP-Q of 0/0/−6, 5/3/8, −3/−3/--. She had no pain during palpation or daily life and the mesh was found without any tension near the right pelvic wall. Thus, the displaced mesh itself most likely did not cause any symptoms. Moreover, she experienced a mixed urinary incontinence. The stress urinary incontinence component disappeared after the repeat sacrocolpopexy, whereas for the OAB she received a Botox injection after 9 months and was asymptomatic afterwards.

Both patients had uneventful postoperative courses 14 and 15 months after surgery, respectively.

## Discussion

Laparoscopic sacrocolpopexy represents the gold standard in advanced prolapse repair [5]. Sacrocolpopexy might be more beneficial than transvaginal mesh surgery in terms of mesh-related complication rates, prolapse recurrence, and de novo dyspareunia [6]. Mesh complications occur infrequently but infections, failure of prolapse repair and mesh erosions necessitate mesh removal and repeat sacrocolpopexy if applicable [7].

However, the management of recurrent pelvic organ prolapse is challenging [5, 8] and data on this issue are scarce [5, 9]. Laparoscopic sacrocolpopexy has proven to be useful in recurrent pelvic organ prolapse after initial vaginal mesh surgery [5, 8] and repeat laparoscopic sacrocolpopexy does not differ from primary laparoscopic sacrocolpopexy or primary vaginal prolapse surgery regarding cure rates, complications, hospital stay, blood loss, and recurrence rates rendering re-do sacrocolpopexy a safe, effective, and feasible treatment option [5, 9, 10].

Laparoscopic sacrocolpopexy confers a low risk of mesh exposure of 0.7–1.4% [2, 11], and up to 8% after 2 and 10.5% after 7 years [7, 12]. The risk is increased in patients where the vagina was opened incidentally during the operation [11], and when the patients have a concomitant total hysterectomy the risk of erosion is up to 27.3%, [13] but no risk factors have been consistently shown to increase mesh erosion rates [12, 14]. In our first patient Gynemesh was used, which is known to induce strong foreign body inflammatory responses with activation of matrix metalloproteinases destroying collagen and elastin [15]. The surgical technique for laparoscopic sacrocolpopexy is hardly standardized, but the use of Amid Class I mesh (monofilament, macroporous) is broadly suggested [1] and titanium-coated polypropylene is sometimes recommended [11] to reduce mesh complications.

A conservative approach with topical estrogen can be attempted to treat mesh erosion, but surgical excision of the eroded mesh is necessary in up to 65.5% [2, 12]. Topical estrogen therapy is frequently ineffective, which is in line with data showing that premenopausal women and women on hormone replacement therapy are at an increased risk for mesh erosion [12].

In our patients the large eroded area required surgical treatment as the posterior vagina was almost completely involved. Recently, Panico et al described four main reasons for laparoscopic sacrocolpopexy mesh failure: mesh detachment from the sacral area, mesh detachment from the vagina, stretched mesh, or no clear cause [5]. We would add mesh erosion to these reasons because in our first patient the large area of erosion required further action and thus resulted in failure of the initial operation, and in both patients it is arguable whether the mesh had detached from the vagina or had never been placed correctly in the first place.

Given the high rate of recurrent prolapse a repeat sacrocolpopexy was carried out in the first patient, placing emphasis on accurate preparation and using a titanized polypropylene mesh. The other patient required repeat sacrocolpopexy owing to failure of the first attempt. The old mesh was removed to reduce the risk for infection and the quantity of implanted mesh, and to minimize the risk for shrinkage and erosion [5].

## Conclusion

Mesh removal after sacrocolpopexy and repeat prolapse surgery can be challenging but is feasible and is aimed at improving patient’s complaints and symptoms.

## Supplementary information

ESM 1 (MP4 103669 kb)

## References

[1] Gluck O, Blaganje M, Veit-Rubin N (2020). Laparoscopic sacrocolpopexy: a comprehensive literature review on current practice. Eur J Obstet Gynecol Reprod Biol.

[2] Baines G, Price N, Jefferis H (2019). Mesh-related complications of laparoscopic sacrocolpopexy. Int Urogynecol J.

[3] Sassani JC, Ross JH, Lopa S (2020). Prolapse recurrence after sacrocolpopexy mesh removal: a retrospective cohort study. Female Pelvic Med Reconstr Surg.

[4] Malekzadeh M, Ramirez-Caban L, Hurtado E (2020). Laparoscopic excision and re-attachment of sacrocolpopexy mesh. J Minim Invasive Gynecol.

[5] Panico G, Campagna G, Vacca L (2022). Redo laparoscopic sacrocolpopexy for POP recurrence: is it the right call?. Eur J Obstet Gynecol Reprod Biol.

[6] Zhang CY, Sun ZJ, Yang J (2021). Sacrocolpopexy compared with transvaginal mesh surgery: a systematic review and meta-analysis. BJOG.

[7] Nygaard I, Brubaker L, Zyczynski HM (2013). Long-term outcomes following abdominal sacrocolpopexy for pelvic organ prolapse. JAMA.

[8] Norinho de Oliveira P, Bourdel N, Rabischong B (2016). What to do with recurrent prolapse after vaginal mesh failure?. J Minim Invasive Gynecol.

[9] Ruess E, Roovers JP, Jeffery S (2020). Management of recurrent pelvic organ prolapse after sacrocolpopexy. A video case series. Int Urogynecol J.

[10] Lallemant M, Grob ATM, Puyraveau M (2022). Comparison of first versus second line sacrocolpopexies in terms of morbidity and mid-term efficacy. Sci Rep.

[11] Campagna G, Pedone Anchora L, Panico G (2020). Titanized polypropylene mesh in laparoscopic sacral colpopexy. Int Urogynecol J.

[12] Kim TY, Jeon MJ (2020). Risk factors for vaginal mesh erosion after sacrocolpopexy in Korean women. PLoS One.

[13] Culligan PJ, Murphy M, Blackwell L (2002). Long-term success of abdominal sacral colpopexy using synthetic mesh. Am J Obstet Gynecol.

[14] Tan-Kim J, Menefee SA, Luber KM (2011). Prevalence and risk factors for mesh erosion after laparoscopic-assisted sacrocolpopexy. Int Urogynecol J.

[15] Liang R, Zong W, Palcsey S (2015). Impact of prolapse meshes on the metabolism of vaginal extracellular matrix in rhesus macaque. Am J Obstet Gynecol.

